# Access to kidney transplantation: outcomes of the non-referred

**DOI:** 10.1186/2047-1440-1-22

**Published:** 2012-12-10

**Authors:** Meteb M AlBugami, Romuald Panek, Steven Soroka, Karthik Tennankore, Bryce A Kiberd

**Affiliations:** 1 Department of Medicine, Dalhousie University, Halifax, NS, Canada 5820 University Avenue, Halifax, NS, B3H 1V8, Canada; 2 Queen Elizabeth II HSC-VG site, Rm. 5082 AC Dickson Building, 5820 University Avenue, Halifax, NS, B3H 1V8, Canada

**Keywords:** Ageism, End stage renal disease, Kidney transplantation, Wait list, Eligibility, Candidacy

## Abstract

**Background:**

There is a concern that some, especially older people, are not referred and could benefit from transplantation.

**Methods:**

We retrospectively examined consecutive incident end stage renal disease (ESRD) patients at our center from January 2006 to December 2009. At ESRD start, patients were classified into those with or without contraindications using Canadian eligibility criteria. Based on referral for transplantation, patients were grouped as CANDIDATE (no contraindication and referred), NEITHER (no contraindication and not referred) and CONTRAINDICATION. The Charlson Comorbidity Index (CCI) was used to assess comorbidity burden.

**Results:**

Of the 437 patients, 133 (30.4%) were CANDIDATE (mean age 50 and CCI 3.0), 59 (13.5%) were NEITHER (age 76 and CCI 4.4), and 245 (56.1%) were CONTRAINDICATION (age 65 and CCI 5.5). Age was the best discriminator between NEITHER and CANDIDATES (c-statistic 0.96, *P* <0.0001) with CCI being less discriminative (0.692, *P* <0.001). CANDIDATES had excellent survival whereas those patients designated NEITHER and CONTRAINDICATION had high mortality rates. NEITHER patients died or developed a contraindication at very high rates. By 1.5 years 50% of the NEITHER patients were no longer eligible for a transplant.

**Conclusions:**

There exists a relatively small population of incident patients not referred who have no contraindications. These are older patients with significant comorbidity who have a small window of opportunity for kidney transplantation.

## Background

One of the most important tasks of a nephrologist is to evaluate and refer patients with End Stage Renal Disease (ESRD) for a kidney transplant. Kidney transplantation provides a better quality of life, on average a longer life and at a lower cost when compared to standard dialysis [1]. Some have argued that many older patients are not being transplanted because of ‘ageism’ [2,3]. In a small prospective study we found that nearly 25% of incident ESRD patients had no contraindications to transplantation using published Canadian consensus recommendations and were not referred. The single most important discriminating factor was age [4]. In those without contraindications to kidney transplantation, patients referred were on average 50 years old whereas those not referred were on average 75 years old.

Details are lacking on the subsequent outcomes of this non-referred cohort that has no contraindication to transplantation. One of the recommendations of the Canadian consensus report on eligibility for transplantation is that patients should have a reasonable probability of surviving beyond the anticipated wait times for a transplant [5]. In this study we sought to further evaluate referral, listing and subsequent outcomes in a larger cohort of patients starting ESRD therapy. Our hypothesis is that there will be a subset of patients not referred who would survive the wait list period free of contraindications if no preemptive kidney was available. Identifying this cohort would be important.

## Methods

The cohort consisted of all consecutive adults (over 18 years of age) initiating ESRD therapy (preemptive transplant, peritoneal or hemodialysis) at this single center from 1 January 2006 to 31 December 2009. In addition, patients with a failed transplant were also included since these would normally be considered eligible for a retransplant. Patients were from mainland Nova Scotia (excluding Cape Breton) but the cohort did not include patients from Yarmouth. There was an overlap with 33 patients from our previous study [4]. The inclusion period of January 2006 was chosen as the start date as this was when electronic health records became available. Follow-up ended 21 March 2011. Permission for the study was obtained from Capital Heath Research Ethics Board. Data Collection: Information on patient age, gender, height, weight, comorbidities, and laboratory (hemoglobin, albumin and creatinine) data at the time of ESRD therapy was extracted from the hospital records. Estimated glomerular filtration rate (eGFR) was calculated using the modification of diet in renal disease (MDRD) formula. The Charlson Comorbidity Index (CCI) was used to quantify comorbidity burden [6]. The CCI is calculated from the presence of comorbid conditions (congestive heart failure, dementia, cancer, cerebral vascular disease, severe renal disease *etcetera*), with each condition being given a weight. The index did not include age. The transplant database was reviewed for evidence of kidney transplantation referral. Candidacy on those referred was determined by the Program’s transplant wait list committee. For patients not referred, decisions about candidacy were made by a consensus of the authors (BK, RP, MA) after independent review of each patient’s electronic records. A decision was made about candidacy at the start of ESRD therapy. For patients without a permanent contraindication a further evaluation was made to determine if and when a permanent contraindication occurred after the start of dialysis. As per our previous paper, contraindications were based on the Canadian Society of Transplantation’s consensus document [5]. Critical contraindications included active angina, acute coronary syndrome, myocardial infarction or stroke within six months, active renal disease (that is, vasculitis on treatment) that could recur in the transplant, severe chronic obstructive lung disease, active peripheral vascular disease, active infection, metastatic cancer or cancer treated within the recommended wait time. Obesity with a body mass index (BMI) greater than 40 kg/m^2^ was also considered a contraindication at our center. Patients (n = 6) who refused the transplant option were classified under contraindication.

Statistical Analysis: Data were presented as means (standard deviation) and by percentages. Differences between groups were tested by parametric and non-parametric tests where appropriate. Patients were categorized as transplant *Candidate* (referred and no contraindication), *Contraindication*, and *Neither* (not referred and no contraindications). Receiver operating curves were used to examine concordance between continuous variables and Candidate *versus* Neither. Multivariable binary logistic analysis was used to examine associations between Candidate and Neither and baseline covariates. Variables included in the analysis were age, gender, diabetes status, CCI (index score and individual comorbidities), laboratory data (albumin, eGFR, hemoglobin), and type of renal disease. A stepwise backward conditional analysis was performed to identify significant predictors.

Patient survival was examined using the Kaplan-Meier method. Log rank was used to detect differences between groups. Survival was censored at last follow-up and at time of transplantation. A Cox hazard model was used to examine variables associated with time to death or time to development of a contraindication in the Neither group. Variables included in the analysis were age, gender, diabetes mellitus status, CCI, laboratory data, and type of renal disease. Individual comorbidities were also examined. A stepwise backward conditional analysis was performed to identify significant predictors. Statistical analysis was performed using SPSS (11.1) software (SPSS Inc. Chicago, IL).

## Results

**Figure 1:**
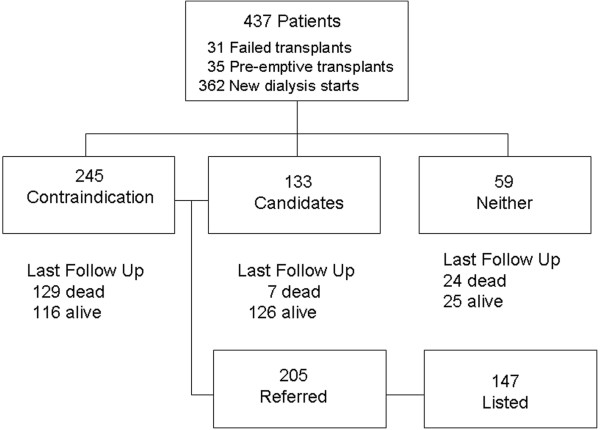
Patient flow.

**Figure 2:**
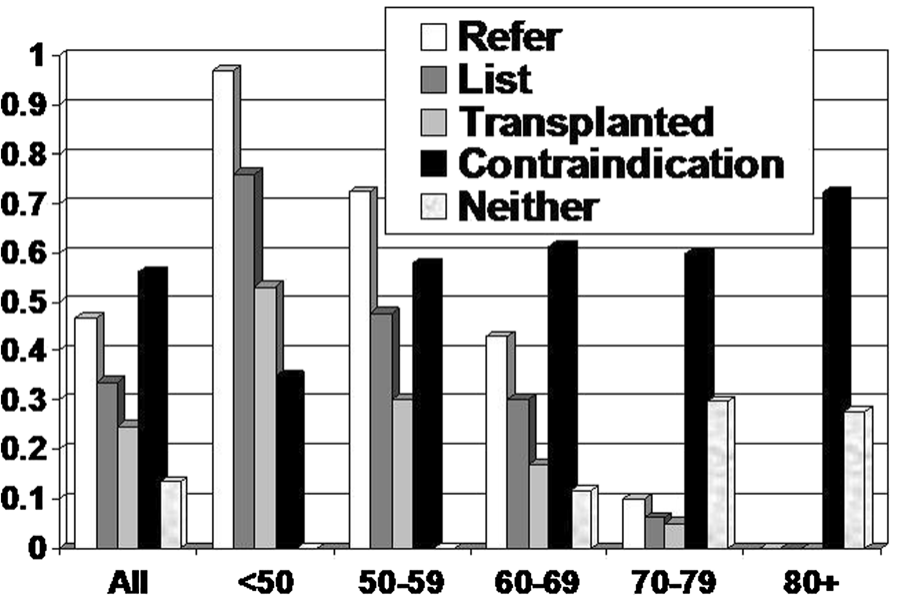
Patient disposition stratified by age.

Of the 437 patients, 31 were those with failed transplants returning to dialysis, 35 received preemptive transplants and the remaining started hemodialysis or peritoneal dialysis. The majority were Caucasian and 3.8% (n = 17) were Black. The mean follow-up time was 2.1 years (median was 1.9 years). Few (n = 2) were lost to follow-up. In all, 205 (47%) were referred for transplantation, 147 (33.6%) were eventually listed, 101 (23.1%) have been transplanted, 160 (36.6%) have died, and initially 245 (56.1%) had contraindications to transplantation. Figure 1 shows the patient flow and Figure 2 shows the breakdown of referral, wait list, transplantation, and initial contraindication by age categories. Fewer older patients were referred and listed. Of those patients age 70 to 79 years, 60% had an initial contraindication, 9.9% (10/111) were referred and 6.3% (7/111) were listed. None of those age 80 plus years were referred and 72% of these had initial contraindications. Of the 245 patients who had an initial contraindication, 72 were referred and 28 were subsequently activated. Those that were eventually referred were between the ages of 25 and 68 years. No patient more than 70 years old with an initial contraindication was later referred, however only three of these older subjects had a reversible contraindication before death.

Based on the initial evaluation at ESRD or return to dialysis in the failed transplants, 133 (30.4%) patients were referred, had no contraindications and were classified as *Candidates*. There were 59 (13.5%) patients without contraindications who were not referred and were categorized as *Neither*. The remaining 245 (56.1%) had a *Contraindication*. Table 1 shows the demographics of these cohorts. The Neither cohort was significantly older than both the other groups and had significantly more comorbidity than the Candidate group as judged by the CCI.

**Table 1:** Baseline patient demographics by cohort

	**All**	**Candidates**	**Neither**	**Contraindication**	**Probability**
	**N = 437**	**N = 133**	**N = 59**	**N = 245**	
Age (years)	62 ± 16	50 ± 14	76 ± 7	65 ± 14	<0.001
Gender male	249 (57%)	72 (54%)	30 (50.4%)	147 (60%)	0.325
ESRD					
DM	121 (28%)	28 (21%)	15 (25%)	78 (32%)	0.311
PCKD	41 (9.3%)	28 (21%)	2 (3.4%)	11 (4.5%)	<0.001
GN	56 (12.8%)	30 (23%)	4 (6.8%)	22 (9%)	0.001
BMI kg/m^2^ n = 368	29.2 ±7.8	28.0 ± 5.9	28.2 ± 5.8	30.1 ± 8.9	0.037
eGFR					
ml/min/1.73 m^2^	8.8 ± 3.8	8.4 ± 3.8	9.2 ± 4.2	9.0 ± 3.7	0.368
Albumin g/L	32 ± 7	35 ± 6	32 ± 5	30 ± 6	<0.001
Hemoglobin g/L	100 ± 15	105 ± 17	103 ± 15	97 ± 18	<0.001
CCI	4.6 ± 2.7	3.0 ± 2.0	4.4 ± 2.3	5.5 ± 2.7	<0.001

*BMI*, body mass index; *CCI*, Charlson Comorbidity Index; *DM*, diabetes mellitus; *eGFR*, estimated glomerular filtration rate; *ESRD*, end stage renal disease; *GN*, glomerulonephritis; *PCKD*, polycystic kidney disease.

**Figure 3:**
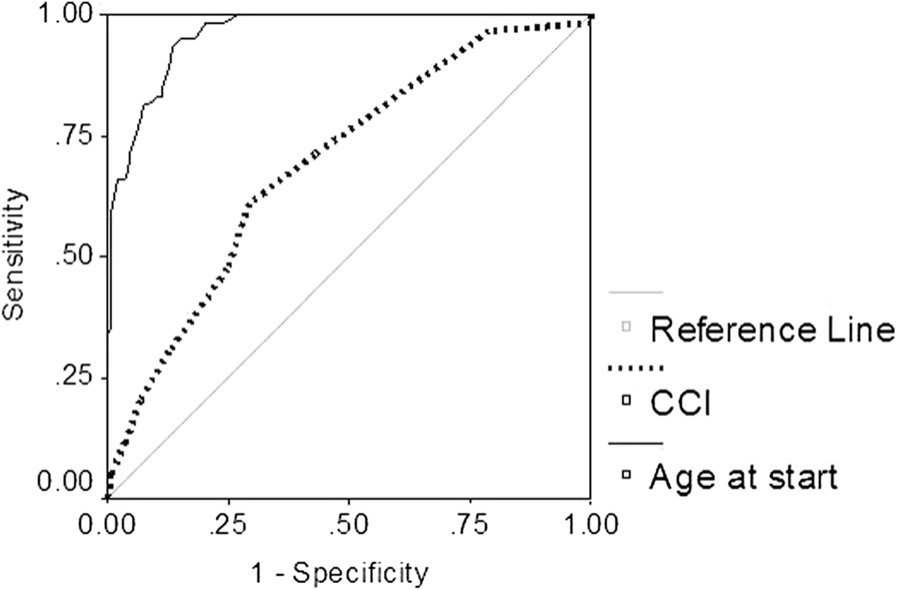
**Receiver operating curve for age and Charlson Comorbidity Index in Candidates *versus *Neither patients.**

**Figure 4:**
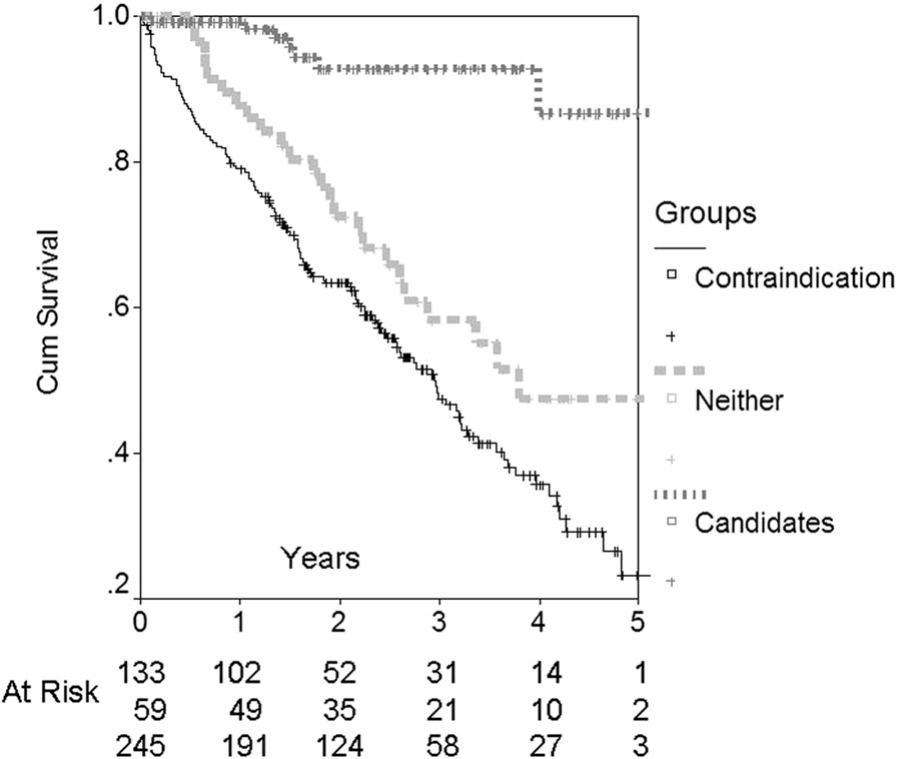
Patient survival based on initial classification.

Among the patients without a contraindication, age was a very significant discriminating variable (Figure 3) differentiating Candidates from Neither patients (c-statistic 0.96, *P* <0.001). Other significant variables were CCI (c = 0.692, *P* <0.001) and albumin (c = 0.659, *P* = 0.002). In a multivariable logistic regression model only age (Odds Ratio (OR) 1.4 per year, 95% CI 1.2, 1.6, *P* <0.001) and CCI (OR 1.5 per unit, 95% CI 1.1, 2.0, *P* = 0.013) were significant variables that distinguished Candidates from Neither. Figure 4 shows patient survival for each of the three cohorts. Neither and Contraindication patients had much higher mortality rates than Candidates (*P* <0.001). Although mortality was higher overall in the Contraindication group compared to the Neither group, the survival curves were essentially parallel after the first three to six months. To emphasize, patient survival was censored at time of transplant and patients with a preemptive transplant were excluded from the survival analysis. Excluding patients with a failed transplant returning to dialysis did not change the findings (data not shown).

**Figure 5:**
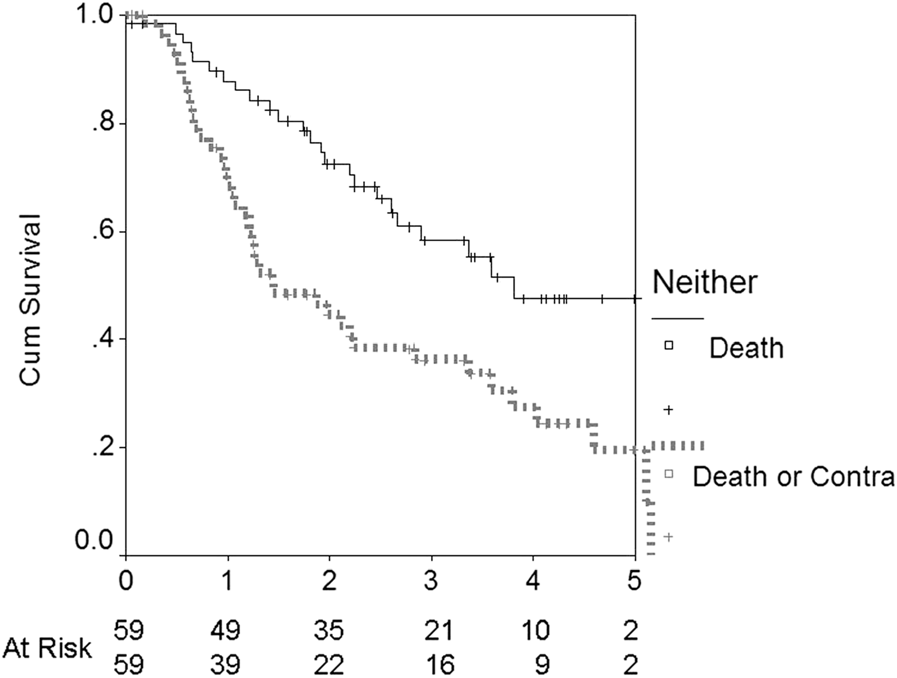
Patient survival and time to Contraindication in the Neither cohort.

In the subset of Neither patients, all were more than 60 years old. Figure 5 shows time to death and time to death or to contraindication Median time to the development of a contraindication or death was only 1.45 years. Reasons for later contraindication were vascular ([7], peripheral, cerebral or coronary artery disease), cancer [6], dementia [4], pulmonary disease [5] and other [6]. In an additional nine subjects, death occurred before any overt complication, leaving only 14 patients who survived the follow-up period without contraindication. In a multivariable Cox analysis only the presence of ischemic heart disease (OR 3.1, 95% CI 1.3, 7.3, *P* = 0.011) was a significant predictor of developing a contraindication. There was a trend for peripheral vascular disease (OR 2.1, 95% CI 0.92, 4.8, *P* = 0.077) to be associated. Age, gender, diabetes status, CCI, other comorbidities, laboratory data and type of renal disease were not predictors.

## Discussion

This study shows that there exists a small subset (<15%) of incident dialysis patients who should be eligible for and might benefit from kidney transplantation. This cohort is older with significant comorbidity and may be shrinking in comparison to the 23% in our report from five years earlier [4]. The unique finding of this study is that this cohort not only has a mortality rate that is similar to patients with transplant contraindications but there is also a very good chance that these patients will develop a contraindication within several years. Other than vascular disease, there is little to predict who would have survived or remain contraindication-free long enough to receive a kidney transplant.

In comparison, our earlier study was prospective whereas this one was retrospective [4]. Although there was a small overlap, all 33 patients were classified the same after a look back. In the current study slightly more were referred (46.6% versus 41.6%), more had initial contraindications (56.1% versus 42.5%) and there were fewer categorized as neither (13.5% versus 23%). It is not clear whether these are significant trends. Surprisingly, the mean ages of the three cohorts were almost exactly the same between the two studies (Candidates 50 versus 50, Contraindications 65 versus 65, and Neither 75 versus 76).

In regard to potentially greater referral, nearly 10% of those patients age 70 to 79 years were referred. None of the incident Candidates were older than 70 years of age in the previous study although the program had older prevalent patients on the wait list in 2006 [4]. Most, but not all, studies have shown that older wait-listed patients have better outcomes with deceased and live donor kidney transplants than they have if remaining on dialysis [8-11]. Studies have also projected that younger recipients would do better to wait for a standard criteria donor organ than to receive an ECD (extended criteria donor) organ if the wait time is not excessive, and older patients would do better to accept an ECD organ quickly rather than run the risk of dying while waiting for a standard criteria organ [12]. Our center allocates ECD donor kidneys preferentially to the older wait list candidates. Given the increasing numbers of older donors, this is an advantage to our older recipients and may have increased our willingness to place older candidates on the list.

In regard to classifying more patients with contraindications, this cohort came from a more restricted catchment area, was retrospective, and we had the benefit of comprehensive electronic health records. This may have helped document the presence or absence of contraindications. The overall CCI (5.5 versus 5.5) in the Contraindication subjects was the same between this and our previous study [4]. Obesity as a sole reason for denying access was observed in only four patients. Patient refusal was observed in seven patients, of which only three were over age 65. One older woman (age 68 years) nearly completed the work-up pre-dialysis and then refused to be listed.

Although the percentage of patients not referred without contraindications is relatively small, it is a substantial proportion (26.5%) of the older people (age 65 and older). Mortality and rates of complications that would result in removal from the wait list are very high. Since transplant wait lists are growing and wait times exceed three years in many centers, only about 3.5% (n = 14) of patients were in retrospect disadvantaged by not having undergone referral. In a recent analysis of US waitlisted patients, almost half of those older than age 60 are likely to die before being transplanted [13]. Annual mortality rates after the second year of waiting increase to more than 25 deaths per 100 patient years in wait-listed candidates older than age 70 [9] There will be additional patients who will have been taken off the list or put on hold. Therefore our Neither (no contraindications and not referred) cohort is likely to have even lower rates of transplantation given competing risks of death and illness compared to the current older wait-listed population.

The study identifies two other subsets of patients who have been disadvantaged. There were six patients with initial contraindications that reversed at a later date and who still have not been referred. All were older than 59 years of age. There were 14 additional patients who had no contraindications but were referred and were never listed. Of these, eight were older than 65 years of age. Reasons for not listing were varied (failure to complete work-up, later refused, death, later contraindication and lost to follow). Of these 20 patients in total, only four have died.

One of the limitations of this study is that not everyone underwent a formal transplant evaluation. This was a retrospective chart review and decisions were made by three individuals, two of whom were part of the earlier study. It is possible the Neither group could be smaller if all underwent a thorough transplant evaluation. On the other hand the group could be larger as other centers might disagree with our decisions. We point out that the mortality in the group with initial contraindications by our interpretation of the Canadian guidelines was very high with a median survival of 2.9 years, and for those with persistent contraindications and who were 60 years or older median survival was only 2.3 years (data not shown). More aggressive listing centers might have seen greater mortality in their Candidates (death on the list) with greater delisting from illness and a smaller Neither group. Less aggressive listing centers might observe lower mortality in those deemed to have Contraindications or they might observe a larger Neither group with better survival.

Another limitation is that the Canadian population has universal health care. US centers with greater diversity and variations in medical insurance coverage may yield different results. The advantage of this study is that the necessary detail to make decisions of this sort may not be possible by examining medical administrative databases. In a large US registry analysis eligibility was retrospectively inferred by examining Medicare claims [14]. Of 128,850 older patients (at least 65 years of age) in this analysis, 11,756 (9.1%) and 43,291 (33.6%) were classified respectively as excellent and good potential candidates. Using their reported percentages, about 5.1% were wait-listed or referred for a live donor transplant. Given differences in methodology and greater scrutiny of the patient’s actual medical records, it is not surprising that our study shows somewhat fewer (26.5%) age 65 years and older patients who were deemed without contraindications (excellent and good potential candidates). However, 13.3% were referred and 8.1% (slightly more) were actually listed. Studies from the United Kingdom and France have also shown that patients age 65 years and older are 93% less likely to be wait-listed compared to younger cohorts [7,15]. In comparison we find that after adjustments for gender and comorbidity, older dialysis patients were 96% (95%, CI 92 to 98%) less likely to be wait-listed compared to those age 50 years and younger. What our study adds is that this subset of not referred without contraindications has a very small window of opportunity (50% are no longer eligible within 1.5 years). The transplant evaluation may well be costly and unproductive for patients in this cohort, especially if there are significant delays in work up. Preemptive live and preemptive listing for expanded criteria deceased donor organs would be necessary if transplantation is to occur. However, the benefits of this approach have not been closely studied. In our study, two patients (age 70 years and older) received preemptive deceased donor transplants and two patients (age 65 years and older) received preemptive live donor transplants.

## Conclusions

Individual centers should scrutinize their referral and listing practice to ensure eligible patients are not missed for kidney transplantation. Patients should be tracked to confirm that ineligible patients have poor outcomes while conversely those who are eligible should survive a reasonable wait time. There exists a population of incident patients that are not referred, who have no obvious contraindications, based on Canadian eligibility criteria and many of these may not survive long enough to receive a transplant before death or contraindication. Better methods of assessment are needed. In addition, the evaluation process should ideally start before dialysis, and greater vigilance is required to consider those who have reversible contraindications or who are lost in the evaluation process.

## Abbreviations

BMI: body mass index; CCI: Charlson Comorbidity Index; DM: diabetes mellitus; ECD: extended or expanded criteria donor; eGFR: estimated glomerular filtration rate; ESRD: end stage renal disease; GN: glomerulonephritis; MDRD: modifications of diet in renal disease; PCKD: polycystic kidney disease.

## Competing interests

The authors declare that they have no competing interests.

## Authors’ contributions

MA determined patient eligibility, collected data, contributed to drafting the paper. RP determined patient eligibility and contributed to drafting the paper. SS collected data and contributed to drafting the paper. KT performed the statistical analysis, collected data, and contributed to drafting the paper. BK designed the study, determined patient eligibility, collected data, performed the statistical analysis, and contributed to drafting the paper. All read and approved the final manuscript.
